# The Effect and Mechanism of Oleanolic Acid in the Treatment of Metabolic Syndrome and Related Cardiovascular Diseases

**DOI:** 10.3390/molecules29040758

**Published:** 2024-02-06

**Authors:** Quanye Luo, Yu Wei, Xuzhen Lv, Wen Chen, Dongmei Yang, Qinhui Tuo

**Affiliations:** 1 Key Laboratory of Vascular Biology and Translational Medicine, Medical School, Hunan University of Chinese Medicine, Changsha 410208, China; 20223777@stu.hnucm.edu.cn (Q.L.); 20223776@stu.hnucm.edu.cn (Y.W.); chenwen@biochen.org (W.C.); 2 Key Laboratory for Quality Evaluation of Bulk Herbs of Hunan Province, The School of Pharmacy, Hunan University of Chinese Medicine, Changsha 410208, China; 20232054@stu.hnucm.edu.cn

**Keywords:** oleanolic acid, metabolic syndrome, cardiovascular diseases

## Abstract

Metabolic syndromes (MetS) and related cardiovascular diseases (CVDs) pose a serious threat to human health. MetS are metabolic disorders characterized by obesity, dyslipidemia, and hypertension, which increase the risk of CVDs’ initiation and development. Although there are many availabile drugs for treating MetS and related CVDs, some side effects also occur. Considering the low-level side effects, many natural products have been tried to treat MetS and CVDs. A five-cyclic triterpenoid natural product, oleanolic acid (OA), has been reported to have many pharmacologic actions such as anti-hypertension, anti-hyperlipidemia, and liver protection. OA has specific advantages in the treatment of MetS and CVDs. OA achieves therapeutic effects through a variety of pathways, attracting great interest and playing a vital role in the treatment of MetS and CVDs. Consequently, in this article, we aim to review the pharmacological actions and potential mechanisms of OA in treating MetS and related CVDs.

## 1. Introduction

Metabolic syndrome (MetS) is a complex cluster of metabolic disorders, including obesity, which increases the risk for the development and progression of cardiovascular diseases (CVDs) [1]. CVDs are deemed to be the main reason for death, including stroke, ischemic cardiomyopathy, and atherosclerosis [2]. Today, many effective medicines have been used in treating MetS and CVDs, such as hydrochlorothiazide and nifedipine, which are used as first-line drugs to reduce blood pressure [3]; metformin is effective in treating hyperglycemia [4]. However, the continuous use of these treatments may cause adverse reactions such as dyspnea, dizziness, vertigo, headaches, and muscle lysis [5,6].

New drugs are needed urgently to treat MetS and related CVDs without significant side effects. Recently, natural products of plant origin attracted the attention of academics because of their potent pharmacological activity and low-level side effects.

The natural product oleanolic acid (OA: 3b-hydroxyolean-12-en-28-oic acid) is a pentacyclic triterpenoid compound. It has been extracted from many species, including *Olea europaea* [7]. Studies on biological activity have shown that OA has a liver-protective effect, and has been listed as a liver-protective drug in China [8]. OA also has anti-inflammatory, anti-oxidant, anti-hyperglycemia, anti-hyperlipidemia, cardioprotective, anti-atherosclerotic, and some other pharmacological effects [9,10,11,12,13].

**Figure 1:**
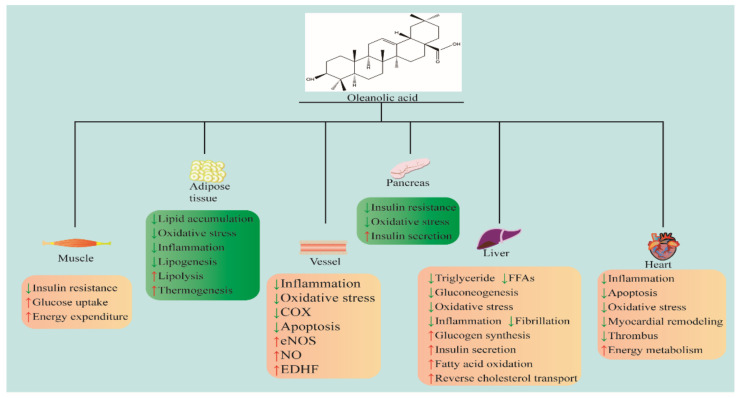
OA in the treatment of MetS and CVDs. Chemical structures of OA drawn according to [14]. COX: cyclooxygenase; eNOS: endothelial nitric oxide synthase; NO: nitric oxide; FFA: free fatty acid; EDHF: endothelium-derived hyperpolarizing factor. ↓ indicates inhibition; ↑ indicates promotion.

Previous research revealed the medicinal value of OA in curing MetS and related CVDs. Consequently, this review summarized the pharmacological effects and mechanisms of OA in treating Mets and related CVDs. Figure 1 summarizes OA in the treatment of MetS and related CVDs.

## 2. Anti-Metabolic Syndromes’ Effects

### 2.1. Anti-Obesity

Obesity is associated with numerous diseases and a shortened life expectancy [15]. Fat production is the maturation of fat cells by which preadipocytes become adipocytes, so they play an essential part in obesity [16]. In the process, CCAAT/enhancer-binding protein (C/EBP) and peroxisome proliferator-activated receptor γ (PPARγ) are thought to be the vital early regulatory proteins for lipogenesis. Adiponectin, sterol regulatory element-binding protein 1 (SREBP1), and fatty acid synthetase (FAS) are in charge of the production of mature fat cells [17]. OA could inhibit the expression of the visceral fat-specific adipokine and downregulate PPARγ and C/EBPα to reduce the intracellular accumulation of fat in adipocytes [18]. Furthermore, OA may reduce obesity via the suppression of the adipogenic factors PPARα, SREBP1, and FAS [19]. OA has been shown to reduce the synthesis of fat and accelerate the utilization of fat through the alteration of hepatic PPARα, recombinant carnitine palmitoyltransferase 1A (CPT1A), SREBP-1, the acetyl coenzyme A carboxylase, and coupled protein 1 (UCP1) [20]. In addition, OA can reduce blood glucose and lipid levels by promoting carbohydrate and fat metabolism [21]. Another piece of research showed that OA can be effective against postmenopausal obesity by inhibiting fat synthesis acetyl-CoA carboxylase (ACC) and upregulating essential genes for estrogen production, CYP11, CYP1, and CYP17A19 [22].

Inflammation is crucial in obesity [23]; chronic inflammation in adipose tissue is primarily driven by macrophages [24] that are classified into two types: M1-type macrophages and M2-type macrophages [25]. An increase in the ratio of M1/M2-type macrophages can enhance adipocyte growth, fat storage, and adipocyte differentiation [26]. Recent research has discovered that OA was able to reduce inflammation via the inhibition of macrophage infiltration, the M1/M2 ratio in adipose tissues, reactive oxygen species (ROS), and decreasing NACHT, LRR, and PYD structural domain protein 3 (NLRP3) [27].

Resistin is an adipocyte-specific secreted factor associated with adipocyte differentiation [28]. OA could reduce resistin synthesis in vivo by stimulating the cellular signaling transcriptional repressor three signaling and interfering with the tyrosine kinase 2-transcriptional signaling sensor activator [29]. Furthermore, glucose homeostasis and adipocyte differentiation are regulated by transcription factor hepatocyte nuclear factor 1b (HNF1b) [30]. The research showed that OA could relieve glucose/lipid metabolic dysfunction via HNF1b [31].

The causes of obesity are complex, the symptoms are diverse, and multiple organs are implicated, so OA in treating obesity is far from sufficient, especially in molecular mechanisms where it is even more insufficient. Therefore, more research is needed to demonstrate the role of OA in treating obesity.

### 2.2. Anti-Hyperlipidemia

Hyperlipidemia is defined as elevations of the fasting total cholesterol concentration, which could directly cause some severe diseases [32]. Numerous studies have suggested that OA is beneficial in the treatment of hyperlipidemia. OA could attenuate the triglycerides (TG) in rats by reducing the fat synthesis factor sterol regulatory element and activating transcription factor 1 [33]. OA also reduces total cholesterol (TC) formation by inhibiting cholesterol acyltransferase activity [34]. A high-fat diet will increase the level of peroxisome proliferator-activated receptor gamma coactivator 1β (PGC-1β) leading to lipogenesis and very-low-density lipoprotein secretion [35]; OA could decrease serum lipids in mice via the inhibition of PGC-1β expression [36]. Clinical investigations also have shown that OA decreased serum lipids in hyperlipidemic patients [37].

Hyperlipidemia is frequently one of the risk factors for various issues. Thus, improving blood lipids is critical for human health. Recent research demonstrated that OA can decrease low-density lipoprotein-cholesterol (LDL-c), TC, and TG in mice. The process is thought to be connected to essential targets of lipid synthesis and accumulation.

### 2.3. Anti-Hypertension

One of the cardiovascular risk factors is hypertension [38]. Research revealed that OA was helpful in hypertension [39,40]. OA could diminish vascular resistance by promoting nitric oxide (NO) and inhibiting COX levels in isolated rat vessels [41]. OA also prevented hypertension in rats via the suppression of NO catabolism [42]. Another study indicated that OA can improve high blood pressure by increasing the expression of eNOS [43]. Meanwhile, OA increased the vasodilator endothelium-derived hyperpolarizing factor (EDHF) and NO to maintain normal blood pressure [44].

The renin–angiotensin system and atrial natriuretic peptide (ANP) are crucial to blood pressure homeostasis [45]. It was found that OA can maintain the homeostasis of blood pressure by inhibiting the renin–angiotensin system and enhancing the fluid balance [46]. OA also could increase the expression of atrial ANP, thus enhancing vascular homeostasis [47]. In addition, the diuretic and nephroprotective properties of OA could reduce hypertension [48]. Furthermore, OA could improve hypertension via upregulating the anti-oxidative stress capacity and enhancing diuretic and natriuretic functions in hypertensive rats [49].

Hypertension is one of the most prevalent systemic metabolic disorders [50]; hypertensive patients also have substantially elevated levels of lipid metabolites [51]. Numerous studies have demonstrated that reducing lipids can improve hypertension. OA was found to reduce hypertension by downregulating the expression of pro-inflammatory factor-secreting phospholipase A2 and fat synthesis factor FAS and inhibiting lipid accumulation [52].

In conclusion, the incidence of hypertension has been rising steadily over the past decade, and the effective treatment of hypertension has a positive impact on middle-age and old-age patients. OA, a natural compound, can protect vascular endothelial cells, enhance body fluid balance, and promote glucose and lipid metabolism to reduce hypertension.

### 2.4. Anti-Nonalcoholic Fatty Liver

Non-alcoholic fatty liver is caused by hepatic steatosis in the liver [53]. Among the pathological mechanisms, the fat overloading in the liver triggered an inflammatory cascade response and subsequently developed into steatohepatitis [54]. Recent research indicated that OA could delay the development of a nonalcoholic fatty liver by reducing inflammation, steatosis, and fibrosis in rats [55]. Furthermore, the liver could be in danger from microbial disorders and increased intestinal permeability, which may exacerbate the inflammatory responses to the nonalcoholic fatty liver [56]; research has shown that OA could treat nonalcoholic fatty liver by ameliorating intestinal barrier dysfunction and the Toll-like receptor 4 (TLR4)-associated inflammatory responses [57].

Oxidative stress induced by a hepatic lipid overload exacerbates liver injury [58]. It was discovered that OA could substantially mitigate a nonalcoholic fatty liver by ameliorating hepatic oxidative stress and decreasing lipid synthesis factor SREBP1 [59].

Liver X receptors (LXR) are highly expressed in the liver and responsible for cholesterol metabolism and homeostasis [60]; LXR primarily activates the hepatic fat synthesis pathway by activating the promoter region of SREBP-1 [61]. Research demonstrated that OA was able to improve the abnormal accumulation of fat in the liver by reducing the expression of LXR and the activity of SREBP-1, as well as increasing the expression of reverse cholesterol transport (RCT)-related genes, including ATP-binding cassette transporter protein (ABC)A1 and ABCG1 [62]. Furthermore, OA could directly inhibit the expression of the SREBP-1 protein and decrease fatty acid accumulation in the body, thus ameliorating the progress of nonalcoholic fatty liver [63].

Briefly speaking, OA inhibits fat accumulation, accelerates cholesterol transport in the liver, and suppresses hepatic inflammation and oxidative stress in the treatment of nonalcoholic fatty liver.

### 2.5. Anti-Diabetes Mellitus

Diabetes mellitus is a metabolic disorder characterized by elevated blood sugar, mainly caused by an absolute or relative insulin deficiency and insulin resistance, classified as type 1 and type 2, with type 2 comprising nearly 95% of cases [64]. Insulin sensitivity can be affected by oxidative stress, inflammation, and metabolic disorders.

Inflammation is significant in diabetes mellitus [65]; an inordinate increase of inflammatory factors hinders insulin receptor signaling and leads to insulin resistance [66]. Research has shown that the expression of TLR4, TLR9, interleukin 6 (IL-6), IL-18, tumor necrosis factor α (TNF-α), TNF-1, and C-reactive protein (CRP) was reduced by OA in diabetic rats [67,68,69,70]. Furthermore, OA also could improve insulin resistance by inhibiting the activity of nuclear factor-κB (NF-κB) [71].

Oxidative stress is closely associated with diabetes and causes deleterious consequences of diabetes [72]. OA could improve the antioxidant capacity in diabetic rats by attenuating the levels of NO and malonaldehyde (MDA), as well as enhancing the level of catalase (CAT) and superoxide dismutase (SOD) [73,74]. In addition, OA was able to enhance the antioxidant function of mitochondria by increasing the expression of glutathione peroxidase 4 (Gpx4) and SOD [75]. Furthermore, OA was reported to improve the mitochondrial ultrastructure and function and antioxidant capacity by inhibiting MDA and ROS levels, as well as increasing CAT, SOD, and glutathione peroxidase (GSH-px) in diabetic rats [11,76,77].

Diabetes is associated with disorders of energy metabolism [78]. Lipid accumulation and the dysregulation of glucose homeostasis are significant causes of insulin resistance [79]. It was demonstrated that OA could improve diabetes by inhibiting the level of α-glucosidase and α-amylase [80]. Meanwhile, OA was able to improve diabetes in rats by stimulating insulin secretion [81] and decreasing blood glucose and blood lipid levels [82], increasing hepatic glycogen and muscle glycogen [83]. The research indicated that OA could prevent hyperglycemia by inhibiting glucose absorption and promoting the change of glucose to glycogen [84]. Elevated blood glucose and glycated hemoglobin (HbA1c) levels (referred to as the prediabetic condition) occurred before the transition from normal to diabetic [85] and OA could improve glucose homeostasis via the reduction of blood glucose and HbA1c levels [86]. It was verified that OA affects diabetes, which was related to increasing glucose transporter-5 (GLUT-5) and GLUT-4 expressions and decreasing FAS and ACC-1 expressions [87]. In addition, OA was observed to maintain glucose homeostasis in rats by decreasing the activity of hexokinase, the expression of glycogen phosphorylase (GP), and increasing the expression of glycogen synthase (GS) [88]. Another study indicated that OA could accelerate glucose and lipid metabolism via increasing the level of PPARγ/α and its related regulators, as well as GLUT-4 and fatty acid transport protein-1 (FATP-1) proteins [89]. Furthermore, takeda G protein-coupled receptor 5 (TGR5) belongs to the g-protein-coupled receptors involved in various cellular physiological effects [90]. By activating the expression of TGR5, OA was able to decrease the blood glucose levels [91]. Based on the accumulated evidence, the imbalance of the phosphatidylinositol-3-kinase (PI3K)/protein kinase B (Akt) signaling pathway could cause the development of diabetes mellitus [92]. OA was verified to inhibit gluconeogenesis by reducing the level of Akt, forkhead box O1 (FoxO1), and glucose-6-phosphatase (G6Pase) [93]. It also exhibited that OA was able to accelerate glucose transport by increasing p-Akt levels and GS levels, as well as decreasing GP levels [94,95]. Furthermore, OA has positive effects on diabetes via increasing PI3K/Akt and AMPK phosphorylation, phosphoenolpyruvate carboxykinase (PEPCK), and G6Pase levels, as well as decreasing the level of the mammalian target of rapamycin (mTOR) [96]. It was discovered that OA could improve insulin resistance through the activation of the level of the insulin receptor substrate (IRS-1) and PI3K/Akt [97]. Moreover, OA may normalize insulin, high-density lipoprotein (HDL), IRS1, GLUT2, GLUT4, and Akt levels, and decrease TC, TG, and low-density lipoprotein (LDL) levels [98]. Furthermore, OA could decrease insulin resistance by improving β-cells [99].

High-glucose environments have been found to cause endothelial cell dysfunction [100]. Research has shown that OA attenuated human umbilical vein endothelial cells (HUVECs) function damage via activating PPARδ, increasing the phosphorylation of Akt and eNOS [101]. Furthermore, persistent hyperglycemia will change blood composition, such as erythrocyte morphology [102], and increase the production of erythropoietin (EPO) [103]. OA could improve diabetes by reducing plasma glucose, HbA1c, and EPO levels and increasing the antioxidant capacity of erythrocytes [104].

Complications caused by diabetes are also a leading cause of harm to human health, such as diabetic nephropathy [105]. Research reported that OA could protect rats against diabetic nephropathy by restoring plasma aldosterone and renal injury molecule-1 [106]. In addition, advanced glycosylation end products, such as renal N-(carboxymethyl) lysine, HbA1c, and glycosylated albumin, are also related to the development of diabetic nephropathy [107]. OA was able to inhibit diabetic nephropathy via a reduction of the level of renal N-(carboxymethyl) lysine, HbA1c, urinary albumin, and urine glycated albumin, as well as increasing the level of plasma insulin and renal creatinine clearance [108]. Furthermore, OA could also restore the damaged renal structure by increasing insulin secretion, renal units, and endothelial-selective adhesion molecules, and decreasing urinary albumin/creatinine levels [109].

There is accumulating evidence that OA cures diabetes by decreasing inflammation, reducing oxidative stress, and protecting endothelial cell function. Furthermore, OA could enhance the glucose–lipid metabolism in diabetic rats, restore blood components damaged by high glucose levels, and alleviate diabetic nephropathy problems. To summarize, OA in the treatment of diabetes mellitus has shown tremendous potential and is supported by numerous pieces of research; however, this research may require additional clinical trials to confirm. The detailed pharmacological effects of OA on metabolic syndrome are shown in Table 1.

## 3. Anti-Cardiovascular Diseases Effects

### 3.1. Anti-Stroke

Stroke is one of the main causes of increased mortality [110], which is affected by inflammation, oxidative stress, and nerve damage [111].

The key mechanism in the formation of ischemic stroke is oxidative stress [112], which also causes neuronal apoptosis, inflammation, and nerve injury [113]. It was reported that OA reduced cerebral ischemic stroke damage by increasing the level of mitochondrial antioxidant α-tocopherol (α-TOC) and GSH, as well as decreasing the leakage of the damage marker lactate dehydrogenase (LDH) [114]. Furthermore, OA was able to improve oxidative stress in brain-injured rats; the results showed that OA treatment significantly increased the activity of SOD, GSH-Px, mitochondrial membrane potential (MMP), and succinate dehydrogenase (SDH), and decreased MDA and LDH levels [115]. Meanwhile, OA also could restrain the blood–brain barrier indicator occludin, matrix metalloproteinase 9 (MMP9), and Evans blue leakage, and inhibit oxidative indicator dihydroethidium fluorescence and MDA expression [116]. In addition, heme oxygenase-1 (HO-1) is the most effective antioxidant response element, and glycogen synthase kinase-3β (GSK-3β) is able to regulate HO-1 in controlling oxidative stress [117]. OA attenuated cytotoxicity and ROS via the regulation of the GSK-3β/HO-1 signal in rats [118].

In general, OA from natural product sources has neuroprotective functions, such as the improvement of the blood–brain barrier, reduction of nerve injury, and cerebral edema in mice; the mechanism was primarily associated with the improvement of oxidative damage. However, it remains to be determined whether OA in stroke treatment has a more promising mechanism.

### 3.2. Heart Protection

Heart disease has a high mortality rate, and the number of deaths is still rising [119]. Oxidative stress is a significant reason for heart disease; the elevated expression of ROS causes cardiomyocyte dysfunction and damage [120]. Research demonstrated that OA promoted the antioxidant capacity of the heart via the reduction level of the lipid peroxidation products [121]. Furthermore, OA was able to prevent diabetic cardiomyopathy through the regulation of HO-1/Nrf2 to increase SOD and GS, as well as decrease MDA and GP [122]. Meanwhile, OA was verified to prevent CVDs by improving the inflammatory reaction, MDA, SOD, GPx, as well as heart weight in rats [123]. In addition, OA could improve myocardial apoptosis by increasing the antioxidant capacity and decreasing apoptosis signaling caspase-3 and BAX activity, increasing Bcl-2 activity [124,125].

Endothelin 1 (ET-1) aggravates the development of CVDs [126], and OA could inhibit cardiomyocyte injury through the regulation of the expression of ET-1 [12]. Furthermore, ET-1 and NF-κB modulate the fibrotic process in the heart, as well as promote the expression of fibronectin in cardiac tissues [127]. OA could improve fibrotic hearts in rats by reducing the activation of NF-κB and ET-1 [128]. Moreover, the Akt/mTOR exacerbates the pathological process of myocardial remodeling [129]; OA performed cardiac protection with the inhibition of vascular remodeling by decreasing the levels of Akt and mTOR [130]. In addition, OA possessed the ability to suppress the platelet aggregation mediated by phospholipase C, thereby aiding in the prevention of cardiovascular thrombosis [131].

Therefore, current research demonstrates that OA could treat a variety of heart diseases, as well as prevent cardiac fibrosis and the cardiac remodeling process. The mechanism includes the inhibition of inflammation, oxidative stress, and the improvement of the expression of vasoconstrictive factors.

### 3.3. Anti-Atherosclerosis

Atherosclerosis (AS) is the underlying pathology of CVDs [132]. OA could prevent AS by inhibiting many pathological developments, such as oxidative stress, endothelial dysfunction, and lipid deposition. Oxidative stress was deemed the critical mechanism in AS [133]. Research demonstrated that OA may safeguard HUVECs damage by inhibiting the levels of lipoprotein receptor 1 (LOX-1), ROS, as well as hypoxia-inducible factor 1 α (HIF-1α) [134]. Moreover, OA has been confirmed to alleviate HUVECs damage via the reduction in the level of ROS and LOX-1, as well as enhancing the level of Nrf2/HO-1 [135].

PPARγ is considered a ligand-activated transcription factor that regulates the glycolipid metabolism, and adiponectin promotes fatty acid biosynthesis and inhibits hepatic gluconeogenesis [136]. OA could reduce lipids and enhance high-density lipoprotein cholesterol (HDL-c) by increasing PPARγ and adiponectin Receptor 1 (AdipoR1) Levels, decreasing AdopoR2 levels [137].

Farnesoid-X-receptor (FXR) is associated with the bile metabolism [138], and angiotensin1-7 (Ang1-7) has been implicated as an AS protector [139]. OA was found to decrease the levels of lipids in rats via the regulation of the expression of FXR and Ang1-7 [13]. In addition, OA inhibited the expression of iNOS, thereby delaying the progression of aortic stenosis [140].

In conclusion, OA can reduce the area of vascular lipid plaque and treat AS by protecting HUVECs, reducing inflammatory factors and the accumulation of lipids. The detailed pharmacological effects of OA on metabolic syndrome-related cardiovascular diseases are shown in Table 2.

## 4. Signaling Pathways of OA for the Treatment of MetS and CVDs

Many pharmacological effects of OA in alleviating Mets have been discovered, such as the inhibition of inflammation, reduction of adipogenesis, and improvement of insulin resistance, and they have also been thoroughly investigated; many of these mechanisms were critical pathways for the treatment of CVDs, such as PPAR, NF-κB, and Akt.

### 4.1. PPAR Signaling Pathway

Peroxisome proliferator-activated receptors (PPARs) belong to the nuclear receptor superfamily, and the PPAR subtypes have been identified, including PPARα, PPARγ, and PPARβ/δ [141]. There is a lot of PPARα expression in brown adipose tissue for promoting energy depletion; PPARγ has high expression levels in white adipose tissue, which could improve energy storage mainly by facilitating adipogenesis and lipotransformation. Meanwhile, PPARγ and C/EBPs are synergistically activated adipogenesis. PPARβ/δ is commonly expressed in skeletal muscle and is involved in fatty acid oxidation [142].

Studies showed that OA could reduce the expression of the visceral fat-specific adipokine adiponectin and the accumulation of fat via the downregulation of PPARγ and C/EBPα [18]. Furthermore, OA could accelerate fat utilization by reducing PPARα, inhibiting CPT1, and promoting UCP1. It was also observed that OA suppressed fat accumulation via the regulation of the level of SERBP1 and ACACA [20].

ChREBP, in combination with SREBP-1, activates adipogenic enzymes, such as ACC and FAS [143]. Research has shown that OA could inhibit SREBP-1 and ChREBP, thus reducing FAS and ACC expression; OA also promoted the expression of PPARα, promoting fat utilization and inhibiting fat production [19]. In addition, OA exhibited an endothelial cell protection effect through the regulation of PPARδ to promote the expression of Akt and eNOS [101]. Adiponectin is regarded as a marker of insulin sensitization [144]. OA can increase GLUT4 and adiponectin protein expression via the activation of PPARγ, and it also can increase the level of fatty acid transport protein 1 (FATP1) and long-chain acyl-CoA synthetase (ACSL) via the activation of PPARα, thereby enhancing insulin sensitivity and accelerating fat utilization [89]. AdipoR1 and AdipoR2 exert beneficial effects on AS; AdipoR1 and AdipoR2 have opposing roles in regulating the glucose and energy metabolism [145]. Studies demonstrated that OA could improve AS in rats by regulating the expression of PPARγ to increase AdipoR1 and decrease AdipoR2 [137].

### 4.2. PI3K/Akt Signaling Pathway

The metabolism of matter usually accompanies the metabolism of energy; the phosphatidylinositol 3-kinase (PI3K)/protein kinase B (PI3K/Akt) signaling pathways are vital for the regulation of growth and metabolism [146]. The PI3K belongs to lipid kinases, whose phosphorylated phosphatidylinositol is an integral part of the membrane of eukaryotic cells [147]; the downstream signaling for the PI3Ks is Akt, and research demonstrated that the PI3K/Akt signaling pathways have an important role in ameliorating diseases such as diabetes, hypertension, and cardiovascular diseases [92].

Research revealed that the Akt signaling pathway is involved in maintaining blood pressure homeostasis [148]. Studies suggested that OA could exert endothelial protective effects primarily via the mediation of PPARδ, activating the Akt and eNOS [101].

The downstream of PI3K/Akt is FoxO1, which regulates hepatic gluconeogenesis [149]. It was verified that OA could inhibit gluconeogenesis by stimulating the phosphorylation of Akt and FoxO1, as well as inhibiting the level of G6Pase [93]. Another piece of research showed that OA protected against diabetes by regulating PI3K/Akt expression to inhibit GSK3 and GS expression, as well as increase GP [94]. In addition, OA may improve insulin resistance via the reduction in the expression of mTOR via PI3K/Akt. OA can also inhibit gluconeogenesis by reducing the expression of G6Pase and PEPCK [96]. IRS signaling is a common pathological mechanism of insulin resistance [150]; OA alleviated insulin resistance by increasing the level of IRS-1, which was achieved by PI3K/Akt [97]. Furthermore, OA also ameliorated insulin resistance by inhibiting the expression of IRS-1 and Akt, as well as GLUT2 and GLUT4 [98].

Myocardial pressure overload stimulates cell surface receptors, resulting in PI3K activation and Akt phosphorylation. Although the acute activation of Akt is cardioprotective, over-activation is detrimental to heart failure [151]. In chronic heart failure, Akt phosphorylation is accompanied by an increased level of FoxO3a phosphorylation [152]. mTOR is upregulated in response to stimuli of cardiac remodeling [152]. GSK3 is another well-established PI3K/Akt signaling pathway target; the hypertrophic myocardium induces the activation of GSK3, resulting in cardiac remodeling [153]. OA revealed potent anti-myocardial hypertrophy and fibrosis activity through the inhibition of Akt activation, which decreased the expression of mTOR, FoxO3a, and GSK3 in rats [130].

### 4.3. NF-κB Signaling Pathway

Nuclear factor-kappa B (NF-κB) is formed by homodimers or heterodimers to coordinate the expression of hundreds of genes. Under physiological conditions, NF-κB is sequestered in the cytoplasm by interacting with any member of the inhibitor-κB (I-κB) family of protein inhibitors, for example, IκBα, IκBβ, and p100. In the presence of activating signals, IκB kinase (IKK) rapidly phosphorylates IκBβ. It promotes IκBα ubiquitination and proteasomal degradation, allowing NF-κB translocated to the nucleus upon activation, where it regulates the transcription of target genes [154].

The TLR signaling pathway is capable of activating NF-κB, which induces an inflammatory reaction [155]. OA could ameliorate inflammation by reducing serum TLR9 and NF-κB [67]. Furthermore, OA could reduce inflammatory cell infiltration by decreasing the level of TLR4/NF-κB in diabetic nephropathy rats [69]. In addition, OA was able to improve inflammation and insulin resistance by reducing TNF-α, IL-6, IRS1, and GLUT4 proteins via NF-κB [71].

**Figure 2:**
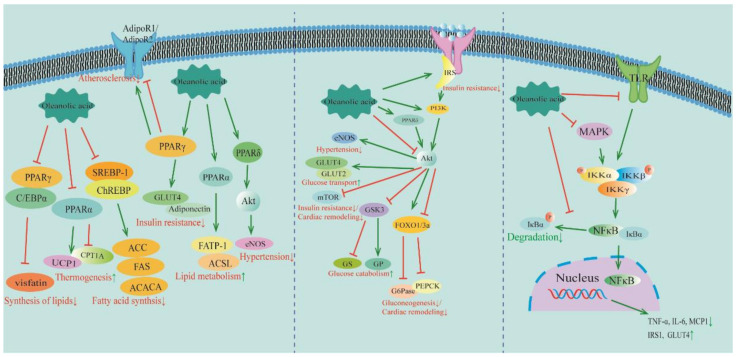
The molecular mechanisms of OA in treating MetS and CVDs: regulation of PPAR, PI3K/Akt, and NF-κB pathways. Information is derived from the references in Table 1 and Table 2. PPAR: peroxisome proliferator-activated receptor; C/EBPα: CCAAT/enhancer-binding protein α; UCP1: coupled protein 1; CPT1A: carnitine palmitoyltransferase 1A; ACC: acetyl-CoA carboxylase; FAS: fat synthesis factor; ACACA: acetyl-CoA carboxylase alpha; GLUT: glucose transporter; FATP-1: fatty acid transport protein-1; ACSL: long-chain acyl-CoA synthetase; Akt: protein kinase B; eNOS: endothelial nitric oxide synthase; IRS: insulin receptor substrate; PI3K: phosphatidylinositol-3-kinase; mTOR: mammalian target of rapamycin; GSK3: glycogen synthase kinase-3; GS: glycogen synthase; GP: glycogen phosphorylase; FoxO1/3a: forkhead box O1/3a; G6Pase: glucose-6-phosphatase; PEPCK: phosphoenolpyruvate carboxykinase; MAPK: mitogen-activated protein kinase; Ikk: IκB kinase; NF-κB: nuclear factor-κB; TNF-α: tumor necrosis factor α; IL-6: interleukin 6; MCP1: monocyte chemoattractant protein-1; GLUT: glucose transporter. ↓ indicates inhibition; ↑ indicates promotion.

The NF-κB and mitogen-activated protein kinase (MAPK) in cardiomyocytes synergistically induce inflammatory responses in cardiac tissues [156]. Research has shown that OA could decrease inflammatory factors such as IL-6, MCP-1, and TNF-α by reducing the level of NF-κB/MAPK [124]. NF-κB also promoted the process of cardiac fibrosis and mediated increased connexin synthesis and the expression of fibrous connexin in myocardial tissues in macrovascular and microvascular endothelial cell lines [157]. OA decreased the expression of IκBβ mRNA and restored the normalization of the expression of down-regulated IκBα mRNA, thereby reducing the level of NF-κB [128]. The molecular mechanisms of OA in treating MetS and CVDs are the regulation of PPAR, PI3K/Akt, and NF-κB, the pathways of which are shown in Figure 2.

## 5. Metabolism, Bioavailability, and Clinical Potential of OA

Pharmacokinetic studies on OA are rarely reported, but are of great interest. Song et al. [158] reported a pharmacokinetic study of OA, made up of 18 healthy Chinese male volunteers receiving a single dose of 40 mg OA in a capsule formulation. The results showed that the peak plasma OA concentration was 12.1 ± 6.8 ng/mL, which appeared at 5.2 ± 2.9 h after the oral administration of OA, and the area under the concentration–time curve (AUC) was 124.3 ± 106.7 ng h/mL. In addition, Rada et al. [159]. conducted a randomized crossover control trial with nine adult male volunteers to study the pharmacokinetics of OA. The volunteers took 70 g of pomace olive oil containing 30 mg of OA. The peak concentrations of OA were reported to be 598.2 ± 176.7 ng/mL, occurring 3.0 ± 0.8 h after administration, with an AUC_0-48_ of 3181.9 ± 894.3 ng h/mL. Additionally, de la Torre et al. [160] conducted a pharmacokinetic investigation of OA involving the administration of 30 mL of olive oil (containing 4.7 mg of OA) to 12 volunteers. The study found that the peak plasma concentration of OA was 5.1 ± 2.1 ng/mL [160]. More recently, there has been a pharmacokinetic study of OA conducted by González et al. [161]. Twenty-two volunteers were administered a single dose of functional olive oil containing 30 mg of OA. The study revealed peak plasma OA concentrations ranging from 500–600 ng/mL, with an AUC_0-∞_ which was 2862.50 ± 174.50 ng h/mL.

OA has a low solubility in water, limiting its own bioavailability and therapeutic potential. Despite the low bioavailability of OA, an intact form of OA is present in tissues after oral intake (4 or 8 weeks) in mice, including the liver and brain. Consequently, these results indicate that OA can cross the blood–brain barrier and have a protective effect on nerves, and the liver was the main organ for the storage and metabolism of OA [162,163]. To address this, there have been some studies combining OA with nanomaterials or using OA as an adjuvant to improve its bioavailability. Férez et al. [164] used cyclodextrins to encapsulate OA, and the results show that OA-encapsulated compounds have advantages over unencapsulated OA in terms of chemical stability, the promotion of cell migration, and the preservation of cell viability. A solidified phospholipid complex composed of an OA–phospholipid complex and hydroxyapatite has been developed, which has increased the oral availability of OA to 240% [165]. Another piece of research developed a self-microemulsifying drug delivery system of OA and conducted pharmacokinetic studies in rats, which increased the bioavailability of OA to 507% [166].

The clinical trials on OA are summarized as follows. Luo et al. [37] designed a small-scale clinical trial to assess the hypolipidemic effect of OA. Hyperlipidemic patients were administrated with OA for four weeks (four tablets at once, three times a day). The results displayed that the TC, TG, and HDL-c levels in the serum decreased significantly. Another clinical study was conducted to assess whether the regular intake of an OA-enriched olive oil is effective in the prevention of diabetes. Prediabetic individuals (176 patients) were randomized to receive 55 mL/day of OA-enriched olive oil (equivalent-dose 30 mg OA/day) or the same oil not enriched (control group). The results showed that the intake of OA-enriched olive oil reduces the risk of developing diabetes in prediabetic patients [167]. Furthermore, a regular consumption of virgin olive oil is associated with a reduced risk of cardiovascular disease. Sanchez-Rodriguez et al. [168] aimed to assess whether the raw intake of a functional olive oil (487 parts per million of phenolic compounds and enriched with 389 parts per million of triterpenes) supplementation (30 mL per day) over three weeks would provide additional health benefits. Fifty-one healthy adults participated in a randomized, crossover, and controlled study. The results showed that the intake of functional olive oil decreased urinary 8-hidroxy-20-deoxyguanosine, plasma IL-8, and TNF-α concentrations. This study provides first-level evidence for the in vivo health benefits, reduction of DNA oxidation, and plasma inflammatory biomarkers of olive oil triterpenoids (oleanolic acid) in healthy humans.

## 6. Conclusions

In summary, OA is a plant-derived pentacyclic triterpenoid natural compound, which has been extensively studied to cure MetS and CVDs in recent years. The research has shown that OA has several therapeutic effects. First, OA decreases blood glucose by increasing insulin sensitivity and promoting glucose uptake and utilization. Second, OA may also inhibit the level of blood lipids and improve the lipid metabolism. In addition, OA has anti-oxidant and anti-inflammatory effects, reducing the injury to the vascular endothelium caused by inflammatory responses. Finally, it also increases NO production, promotes vasodilation, reduces blood pressure, and prevents AS. Overall, OA has various beneficial effects as a natural product for MetS and related cardiovascular diseases.

However, OA also has some objective limitations in the treatment of MetS and CVDs. First of all, although OA is considered a natural and safe drug or food supplement, there are few studies focusing on the toxic side effects of OA, which should be concerned in the future. Secondly, OA has low bioavailability and is insoluble in water. But, there have already been some studies on the combination of OA with nanomaterials, or as an adjuvant to improve the bioavailability of OA, which have achieved good results. Finally, there have been a few clinical trials on OA. There are problems concerning clinical trials such as the small number of participants, the uncertainty of molecular targets, and the short experimental cycle, and preclinical experiments should be combined to determine the efficacy, molecular targets, and safety of OA.

## Figures and Tables

**Table 1:** Pharmacological Effects of OA in the Treatment of MetS.

Experimental Models	Dose of OA	Signaling Pathways	Pharmacologic Action	Refs.
Obesity				
3T3-L1 cells	1 to 25 μM/L OA for 6 days	↓PPARγ, ↓C/EBPα, ↓adiponectin	↓Lipid accumulation	[18]
Female C57BL/6J mice induced by high-fat diet (HFD)	OA in water feeders at 0.005% for 16 weeks	↑↑CD36, ↑PPARα, ↑↑SREBP1, ↓↓FAS	↓↓Adipose tissue weights, ↓↓↓TG	[19]
HFD-induced C57BL6/J male mice	300 mg/kg OA for 10 weeks	↓PPARα, ↓↓CPT1A, ↓SERBP1, ↑↑UCP1	↓↓TC, ↓↓LDL, ↑↑HDL, ↓VLDL	[20]
HFD-induced male Swiss mice	5, 10, or 20 mg/kg OA for 7 days	↑Blood glucose tolerance	↓Plasma lipids, ↓blood glucose	[21]
3T3-L1 cells	3 μg/mL OA for 16 days	↓ACC, ↑CYP11A1, ↑↑CYP17, ↑↑CYP19, ↓↓CYP1A1	↓↓Fat production, ↑estrogen homeostasis	[22]
C57BL/6J mice were fed with HFD	25 and 50 mg/kg OA for 4 weeks	↓↓↓ROS, ↓NLRP3	↓↓↓Adipose tissue hypertrophy	[27]
3T3-L1 cells	1 to 25 μM OA for 2 days	↓STAT1/3, ↓Tyk2, ↑SOCS3, ↓resistin	↓Adipogenesis	[29]
Polychlorinated biphenyls-induced male C57B6/J mice	50 mg/kg OA for 10 weeks	↑HNF1b, ↓ROS, ↓NOX4, ↑SOD1/2, ↑GPx1	↓TG, ↓TC, ↓FFAs, ↓adipocyte size	[31]
Hyperlipidemia				
HFD-male Sprague-Dawley (SD) rats	50 mg/kg OA for 4 weeks	↓↓Levels of acetyl-CoA carboxylase, ↓↓glycerol-3-phosphate acyltransferase, ↓↓Srebf1	↓TG, ↓TC, ↓phospholipid	[33]
Human colorectal adenocarcinoma cells and typical western-diet-induced male Lakeview Golden Syrian hamsters	50 μg OA in vitro; 0.01% OA for 4 weeks in vivo	↓Enzyme cholesterol acyltransferase activity	↓VLDL, ↓LDL, ↓TC	[34]
Male C57BL/6 mice were fed HFD	20 mg/kg for 4 weeks	↓↓PGC-1β	↓↓TG, ↓↓TC, ↓↓LDL-c	[36]
Patients with hyperlipidemia	OA 4 tablets once, three times a day for 4 weeks	↑↑↑CACNA1B, ↓FCN, ↑STEAP3, ↑AMPH, ↑NR6A1	↓TC, ↓TG, ↓HDL-c	[37]
Hypertension				
Male spontaneously hypertensive rats (SHR)	10^−7^ to 10^−4^ M OA	↑NO	↑↑↑Vasorelaxation	[39]
Male Wistar and Dahl salt-sensitive rats induced by a high-salt Na^+^ diet	160 μM OA	↑NO, ↓COX	↑↑↑Relaxation in aortic rings	[41]
Dexamethasone-induced male Wistar rats	60 mg/kg for 5 days	↑Plasma nitrate/nitrite, ↑NO	↓↓Systolic pressure	[42]
HFD-induced Wistar Kyoto rats and SHR	800 parts per million OA for 12 weeks	↑eNOS	↑↑↑Relaxation aorta	[43]
Male Wistar Kyoto rats, and HFD-induced SHR	800 parts per million OA for 12 weeks	↑NO/EDHF	↓↓Endothelial dysfunction	[44]
Male SD rats induced by two-kidney, one-clip hypertensive	20 and 30 mg/kg/day OA for 7 days	↓↓Renin activity, ↓↓angiotensin II type-1/2 receptor, ↓aldosterone, ↑↑↑ANP	↑↑↑Glomerular filtration rate, ↑↑↑electrolyte excretion, ↑↑↑urinary volume, ↓↓↓arterial blood pressure	[46]
Isoproterenol-induced male SD rats	10, 20, or 30 mg/kg/day OA for 2 weeks	↑↑↑ANP	↓Atrial pressure, ↓pulse pressure	[47]
Glucocorticoid-induced male Wistar rats	60 mg/kg/day OA for 4 weeks	↑↑Urine volume, ↑↑urine sodium, ↑potassium	↓↓↓Blood pressure	[48]
Dahl salt-sensitive genetically hypertensive rats and normotensive Dahl salt-resistant rats	60 mg/kg OA for 6 weeks	↑GPx, ↑SOD	↑Systolic and diastolic blood pressure	[49]
SHR and Wistar Kyoto rats	1.08 mg/kg OA for 4 weeks	↓FAS, ↓sPLA2	↓↓TG, ↓LDL-c, ↓↓systolic blood pressure and diastolic blood pressure	[52]
Nonalcoholic fatty liver				
Fructose-induced male and female SD rats	60 mg/kg for 7 days	↓Inflammation, ↓steatosis and fibrosis	↓↓Body mass, ↓liver mass, ↓hepatic lipid storage	[55]
HFD with 60 kcal% fat-induced rats	25, 50, or 100 mg/kg for 8 weeks	↓↓IL-6, ↓↓TLR4, ↓↓IL-1β, ↓↓TNF-α	↓↓↓Body weight, ↓↓↓fatty liver score, ↓↓fasting blood glucose, ↓↓TG, ↓↓TC, ↓ALT, ↓AST	[57]
Male SD rats induced by a high-fat high-carbohydrate diet	80 mg/kg for 12 weeks	↓SREBP1, ↓MDA, ↑GPX, ↑SOD	↓Body/liver weight ratio, ↓TG, ↓VLDL, ↑total bilirubin, ↓ALT, ↓AST	[59]
HepG2 cells	5, 10, or 20 μM OA	↓↓↓LXR, ↓↓↓SREBP-1c, ↑↑↑ABCA1, ↑↑↑ABCG1	↓↓↓Lipogenesis	[62]
Liquid fructose-induced male SD rats	5 or 25 mg/kg OA for 10 weeks	↓SREBP-1	↓TG, ↓lipid accumulation	[63]
Diabetes mellitus				
Male SD rats induced by Streptozotocin (STZ)	5 mg/kg OA for 21 days	↓↓↓TLR9, ↓↓↓NF-κB, ↓IL-18, ↓↓↓MDA	↓↓Glucose	[67]
High-fructose diet in male SD rats and pups	60 mg/kg OA for 14 days	↓↓↓TNF-α, ↓↓↓IL-6, ↑↑↑MAPK, ↑↑↑adiponectin	↓Diabetes	[68]
Male SD rats induced by STZ and a high-fat diet	25 or 100 mg/kg OA for 8 weeks	↓↓TLR4, ↓↓NF-κB	↓↓Fasting blood glucose	[69]
Male SD rats induced by high-fat and high-carbohydrate diet	80 mg/kg OA for 12 weeks	↓TNF-α, ↓IL-1β, ↓CRP	↓Diabetes, ↓immune cell counts	[70]
HepG2 cells induced by free fatty acids	5, 10, or 25 μM/L OA for 24 h	↓↓NF-κB, ↓↓IL-6, ↑↑IRS1, ↑GLUT4, ↓↓TNF-α	↓Insulin resistance, ↓blood glucose	[71]
Male SD rats induced by high-fat and -fructose (HFF) diet	25 mg/kg OA for 6 weeks	↓MDA, ↓NO, ↑SOD, ↑CAT	↓Body weights, ↓serum insulin	[73]
STZ and high sugar and fat-induced female SD rats	25 mg/kg OA for 6 weeks	↓↓↓MDA, ↓↓↓NO, ↑↑↑SOD, ↑↑↑CAT	↓↓↓Weight gain, ↓↓↓fasting blood glucose levels, ↑↑↑insulin sensitivity index	[74]
STZ-induced male SD rats	100 mg/kg OA for 4 weeks	↑GPx, ↑SOD	↓Blood glucose, ↑body weight	[75]
HFD-induced Wistar rats	60 or 100 mg/kg OA for 40 days	↓↓MDA, ↑SOD, ↑↑GSH-px	↓↓Blood glucose	[76]
C57BL/KsJ-Lepdb (db/db) mice and wild mice	20 mg/kg/day OA for 2 weeks	↓ROS, ↑Nrf2	↓Fasting blood glucose	[11]
STZ-induced male SD rats	20, 40, or 60 mg/kg/day OA for 8 weeks	↑CAT, ↑↑↑SOD, ↑GSH	↓Diabetes	[77]
Bioactive compound(s)	Not mentioned	↓α-glycosidase, ↓α-amylase activities	↓Diabetes	[80]
Glucose-pancreatic β-cells, rat islets	30 or 50 μM OA	↑↑Insulin secretion	↓Blood glucose	[81]
STZ-induced male Wistar rats	100 or 200 mg/kg OA for 40 days	↑↑Insulin	↓↓Blood glucose, ↓↓blood lipids	[82]
STZ-induced male SD rats	40, 80, or 120 mg/kg OA for 5 weeks	↑Hepatic glycogen, ↑muscle glycogen	↓Blood glucose, ↑insulin sensitivity	[83]
STZ-induced male Wistar rats	80 mg/kg OA for 18 h	↓Glucose uptake	↓Blood glucose	[84]
Male SD rats induced by a high-fat high-carbohydrate diet	80 mg/kg OA for 12 weeks	↓HbA1c	↓Caloric intake, ↓body weight, ↓blood glucose	[86]
High-fructose-diet-induced SD rats	60 mg/kg OA for 7 days	↑↑Nrf-1, ↓Acc-1, ↑↑GLUT-4, ↓FAS, ↑GLUT-5	↓↓↓Body mass, ↓visceral fat	[87]
STZ-induced male SD rats	80 mg/kg OA for 14 days	↓GP, ↓GS, ↓hexokinase activity	↑Glycogen homeostasis	[88]
C2C12 muscle cells and 3T3-L1 cells	1 to 50 μM OA	↑PPARγ/α, ↑GLUT4, ↑FATP1	↑Lipid homeostasis	[89]
HFD-induced male C57BL/6J mice	100 mg/kg/day OA for 7 days	↑TGR5	↓Serum glucose, ↓insulin levels	[91]
STZ-induced male C57BL/6J mice were fed HFD	100 mg/kg/day OA for 2 weeks	↑p-Akt, ↑↑p-FoxO1, ↓G6Pase	↓↓Urine glucose, ↓↓gluconeogenesis	[93]
STZ-induced male SD rats	100 mg/kg OA for 14 days	↑p-Akt, ↑GS, ↓GP	↓Blood glucose	[94]
Male C57BL/KsJ-Lepdb (db/db) mice	250 mg/kg OA for 4 weeks	↑Akt, ↑PI3K, ↑AMPK, ↓↓↓G6Pase, ↓mTOR, ↓↓↓PEPCK, ↓GP	↓↓↓Blood glucose, ↑gluconeogenesis	[96]
High-fructose-induced male SD rats	25 mg/kg/day OA for 10 weeks	↑IRS-1, ↑PI3K, ↑p-Akt	↓Plasma glucose	[97]
STZ-induced male Institute of Cancer Research mice	25, 50, or 75 mg/kg OA for 15 days	↑↑IRS1, ↑↑GLUT2, ↑↑GLUT4, ↑↑Akt	↓TC, ↓↓TG, ↓↓LDL, ↑↑HDL, ↓↓blood glucose	[98]
Fructose-induced male and female SD rats	60 mg/kg OA for 7 days	↓β-cell dysfunction	↓Insulin resistance	[99]
High-glucose-induced human vascular endothelial cells	0.1 to 50 μM OA for 24 h	↑PPARβ/δ, ↑eNOS, ↑p-eNOS, ↑p-Akt	↓Endothelial dysfunction	[101]
STZ-induced male SD rats	80 mg/kg OA for 5 weeks	↓HbA1c, ↓EPO, ↓MDA, ↑SOD, ↑GPx	↓Diabetes	[104]
High-fat-high-carbo-hydrate-diet-induced male SD rats	80 mg/kg OA for 12 weeks	↓Aldosterone, ↓KIM-1	↓Blood and urine electrolytes, ↓estimated glomerular filtration rate, ↓albumin/creatinine ratio	[106]
STZ-induced male Balb/cA mice	0.05, 0.1, or 0.2% OA for 10 weeks	↓HbA1c, ↓fructose,↓renal N^ε^-(carboxymethyl)lysine	↓Plasma glucose, ↑plasma insulin	[108]
Otsuka Long-Evans Tokushima fatty rats	100 mg/kg OA for 20 weeks	↓Urinary albumin/creatinine levels	↑Blood insulin secretion, ↓ER stress, ↓damaged kidney structures	[109]

The number of arrows indicates different statistical significance: ↓/↑: *p* < 0.05; ↓↓/↑↑: *p* < 0.01; ↓↓↓/↑↑↑: *p* < 0.001. PPAR: peroxisome proliferator-activated receptor; C/EBPα: CCAAT/enhancer-binding protein α; CD36: cluster of differentiation 36; SREBP1: sterol regulatory element-binding protein 1; FAS: fatty acid synthetase; CPT1A: carnitine palmitoyltransferase 1A; UCP1: coupled protein 1; TC: total cholesterol; TG: triglycerides; LDL-c: low-sensitivity lipoprotein-cholesterol; HDL-c: high-density lipoprotein-cholesterol; VLDL: very-low-density lipoprotein; ACC: acetyl-CoA carboxylase; ROS: reactive oxygen species; NLRP3: NACHT, LRR, and PYD structural domain protein 3; STAT: signal transducers and activators of transcription; Tyk2: tyrosine kinase 2; SOCS3: suppressor of cytokine signaling 3; HNF1b: hepatocyte nuclear factor 1b; Nox4: nicotinamide adenine dinucleotide phosphate oxidase 4; SOD: superoxide dismutase; Gpx1: glutathioneperoxidase; FFA: free fatty acid; PGC-1β: peroxisome proliferator-activated receptor gamma coactivator 1β; NO: nitric oxide; COX: cyclooxygenase; eNOS: endothelial nitric oxide synthase; EDHF: endothelium-derived hyperpolarizing factor; ANP: atrialnatriureticpeptide; sPLA2: secretory phospholipase A2; IL-6: interleukin 6; TLR4: toll-like receptor 4; IL-1β: interleukin 1β; TNF-α: tumor necrosis factor α; MDA: malonaldehyde; LXR: Liver X receptors; ABCA/ABCG1: ATP-binding cassette transporter A1/G1; NF-κB: nuclear factor-κB; CRP: C-reactive protein; IRS1: insulin receptor substrate 1; GLUT4: glucose transporter 4; CAT: catalase; GSH-px: glutathione peroxidase; Nrf2: nuclear factor erythroid-2-related factor 2; HbA1c: glycated hemoglobin; GP: glycogen phosphorylase; GS: glycogen synthase; FATP1: fatty acid transport protein 1; TGR5: takeda G protein-coupled receptor 5; PI3K: phosphatidylinositol-3-kinase; Akt: protein kinase B; FoxO1: forkhead box O1; G6Pase: glucose-6-phosphatase; mTOR: mammalian target of rapamycin; PERCK: phosphoenolpyruvate carboxykinase; KIM-1: kidney injury molecule 1; ER: endoplasmic reticulum; ALT: alanine aminotransferase; AST: aspartate transaminase.

**Table 2:** Pharmacological effects of OA in the treatment of CVDs.

Experimental Models	Dose of OA	Signaling Pathways	Pharmacologic Action	Refs.
Stroke				
Ischemia reperfusion experiment-induced female SD rats	0.6 or 1.2 mM/kg OA for 3 days	↓LDH, ↑GSH, ↑α-TOC	↓Brain injury	[114]
Male Institute of Cancer Research mice and male SD rats injured by bilateral common carotid artery ligation	25 or 50 mg/kg OA for 4 days	↑SOD, ↑↑GSH-Px, ↓↓MDA, ↓↓LDH, ↑MMP, ↑↑SDH	↑↑Survival time, ↓cerebral infarction area	[115]
Male Institute of Cancer Research mice	6 mg/kg/day OA for 3 days	↓Evans blue leakage, ↓MMP9, ↓occludin, ↓dihydroethidium fluorescence, ↓MDA	↓Infarct volumes, ↑locomotor activity, ↑memory ability	[116]
SH-SY5Y Cells and rats	10, 20, or 40 μM OA for 12 h in vitro; 10 or 20 mg/kg OA for 3 days in vivo	↑↑GSK-3β, ↑↑HO-1, ↓↓ROS	↓↓Infarct volume in the brain, ↓↓apoptosis	[118]
Heart protection				
Isoproterenol-induced adult male albino rats of the Wistar strain	20, 40, or 60 mg/kg OA for 7 days	↓ALT, ↓AST, ↓CPK, ↓LDH, ↓TBARS	↑Heart protection	[121]
STZ-induced male SD rats	80 mg/kg OA for 14 days	↓↓↓GS, ↓↓↓GP, ↑↑↑HO-1, ↑↑↑Nrf2	↓↓↓Diabetic cardiomyopathy	[122]
SD rats to high-fat high-carbohydrate diet	Not mentioned	↓CRP, ↓IL-6, ↓TNF-α, ↓MDA, ↑SOD, ↑GPx	↓Mean arterial pressure, ↓heart weights, ↓TG, ↓TC, ↓LDL-c, ↓HDL-c	[123]
H9c2 cells	5 or 10 μM OA	↓ROS, ↓GSSG, ↓IL-6, ↓TNF-α, ↑GSH, ↑GPX, ↑GR, ↑CAT, ↑NF-κB, ↑caspase-3, ↓bcl-2	↑Cell viability, ↓plasma membrane damage, ↓apoptosis	[12]
High-glucose-induced H9c2 cells	20 or 50 μM OA for 6 and 20 h	↓↓↓Caspase-3, ↑↑SOD, ↓ROS	↓↓↓Apoptosis, ↑↑↑heart protection	[125]
High-glucose-induced injury in neonatal rat ventricular cardiomyocytes	10 μM OA for 24 h	↓↓↓BNP, ↓↓ET-1, ↓↓MMP	↓↓Cardiomyocyte damage	[12]
Male Zucker Diabetic fatty rats and lipopolysaccharide-induced RAW264.7	Not mentioned exactly in vivo; 10 to 300 mM OA for 24 h in vitro	↓↓ET-1, ↓ETA, ↓IκBβ, ↑↑IκBα	↓↓Cardiac fibrosis	[128]
C57BL/6J male mice and H9c2 cells	25 or 100 mg/kg/day OA for 8 weeks	↓Akt, ↓mTOR, ↓GSK-3β, ↓FoxO3a	↓Cardiac hypertrophy, ↓tissue fibrosis	[130]
The platelets	25, 50, 100, or 200 μM OA	↑↑Phospholipase C	↓↓Platelets aggregation	[131]
Atherosclerosis				
HUVECs induced by Ox-LDL	1, 5, or 10 μM OA	↓LOX-1, ↓ROS, ↓HIF-1α	↓Apoptosis	[134]
High-fat-diet-induced male quails and HUVECs induced by Ox-LDL	25, 50, or 100 mg/kg OA for 10 weeks in vivo; 5, 10, or 20 μM OA for 24 h in vitro	↓NADPH, ↓ROS, ↑Nrf2, ↑HO-1, ↓LOX-1	↓TG, ↓TC, ↓LDL, ↑HDL	[135]
New Zealand rabbits and C57BL/6J mice and Apoe-/- mice fed with an atherogenic diet	25 mg/kg OA for 5 weeks	↑PPARγ, ↑↑AdipoR1, ↓↓AdipoR2	↓↓TG, ↓TC, ↓↓LDL-c,↓intimal thickening of the artery	[137]
Atherogenic diet (1% cholesterol and 5% lard oil)-induced male New Zealand White rabbits and Ox-LDL-induced HUVECs	50 mg/kg/day OA for 28 days in vivo; 40 μM OA for 24 h in vitro	↑↑↑Ang1-7, ↑↑↑NO, ↑↑↑eNOS, ↑FXR	↓↓↓TC, ↓↓↓TG, ↓LDL-C, ↓↓HDL-C, ↓↓↓cell apoptosis, ↓intimal thickening of the artery	[13]
ApoE-/- mice were fed a high-cholesterol Western-type diet	100 mg/kg/day OA for 8 weeks	↓iNOS	↓Plaque area, ↓↓TC, ↓↓plaque area	[140]

The number of arrows indicates different statistical significance: ↓/↑: *p* < 0.05; ↓↓/↑↑: *p* < 0.01; ↓↓↓/↑↑↑: *p* < 0.001. LDH: lactate dehydrogenase; GSH: glutathione; α-TOC: α-tocopherol; SOD: superoxide dismutase; GSH-px: glutathione peroxidase; MDA: malonaldehyde; MMP: mitochondrial membrane potential; MMP9: matrix metalloproteinase 9; GSK-3β: glycogen synthase kinase-3β; HO-1: heme oxygenase-1; ROS: reactive oxygen species; ALT: alanine aminotransferase; AST: aspartate transaminase; CPK: creatine phosphokinase; TBARS: thiobarbituric acid reactive substances; Nrf2: nuclear factor erythroid-2-related factor 2; CRP: C-reactive protein; IL-6: interleukin 6; TNF-α: tumor necrosis factor α; Gpx: glutathione peroxidase; TG: triglycerides; TC: total cholesterol; LDL-c: low-density lipoprotein-cholesterol; HDL-c: high-density lipoprotein-cholesterol; GSSG: oxidized glutathione; GR: glutathione reductase; CAT: catalase; NF-κB: Nuclear factor-κB; Bcl-2: B-cell lymphoma-2; BNP: natriuretic peptide B; ET-1: endothelin 1; MMP: mitochondrial membrane potential; ETA: endothelin receptors; IκBβ/α: inhibitory protein β/α; Akt: protein kinase B; mTOR: mammalian target of rapamycin; GSK-3β: glycogen synthase kinase-3β; FoxO3a: forkhead box class O3a; LOX-1: lipoprotein receptor 1; HIF-1α: hypoxia-inducible factor 1 α; NADPH: nicotinamide adenine dinucleotide phosphate; PPAR: peroxisome proliferator-activated receptor; AdiPoR1/2: adiponectin Receptor 1/2; Ang1-7: angiotensin 1-7; NO: nitric oxide; eNOS: endothelial nitric oxide synthase; FXR: farnesoid-X-receptor; iNOS: inducible nitric oxide synthase.
